# Prevalence and Correlational Factors of Suicidal Ideation and Suicide Attempts Among Chinese Adolescents

**DOI:** 10.3389/fpsyg.2022.911502

**Published:** 2022-06-14

**Authors:** Yan Yan, Xiaosong Gai

**Affiliations:** School of Psychology, Northeast Normal University, Changchun, China

**Keywords:** suicidal ideation, suicide attempts, correlational factors, adolescents, a large-sample survey

## Abstract

This study’s purpose was to (1) determine the prevalence of suicidality (i.e., suicidal ideation or suicide attempts) among adolescents in a city in Northeast China and (2) identify the correlational factors among adolescents with suicidality. A total of 69,519 adolescents from grades 5 to 12 in a city in Northeast China participated in the online investigation. Students completed a structured questionnaire to report their demographic information, psychological characteristics, and suicidality. Univariable and multivariable logistic regressions were applied to determine significant correlational factors associated with suicidal ideation (SI) and suicide attempts (SA). The prevalence of SI and SA among adolescents in the past 12 months was 13 and 4.8%, respectively. Multivariable logistic regression analyses found that the potential risk factors for SI and SA included female, non-nuclear family, higher subjective socioeconomic status, meaningless in life, depression, bullying perpetrator, negative parental rearing styles, lower self-esteem scores, hopelessness, and stressful life events. In order to improve the accuracy of suicide risk identification, a cumulative risk index was used. With the increase in the number of cumulative risks, the risk of SI and SA also increased. So the cumulative risk index was very valuable. The total prevalence of SI and SA among primary and middle school students was high. Preventive measures could be implemented according to the risk factors.

## Introduction

Suicide is one of serious public problems in China and around the world (Franklin et al., 2017; O’Connor and Portzky, 2018). In 2019, there were an estimated 700,000 suicide deaths worldwide and more than 116,324 people die due to suicide in China, suicide occurs throughout the lifespan and was the fourth leading cause of death among 15–29 year olds globally (World Health Organization, 2019). More than 10,000 young people in China die of suicide each year; it is the leading cause of death among people aged 15–34, accounting for 19% of all deaths (Parry, 2014). Youth suicidal thoughts and behavior are an important public health concern (Hawton and O’Connor, 2012; Nock et al., 2013; Felez-Nobrega et al., 2020). Every suicide is a tragedy that affects families, communities, and entire countries, and each one has long-lasting effects on the people left behind (Wang et al., 2019). Therefore, it is imperative to strengthen suicide prevention interventions targeted at adolescents in China.

Suicidal behaviors include suicidal ideation, suicide attempts, and suicide (Sveticic and De Leo, 2012; Du and Jiang, 2015). Suicidal ideation (SI) refers to various intentions to threaten one’s own life, which include observable behaviors, but does not result in suicidal actions. It is characterized by concealment, extensive contingency, and individual differences and often precedes suicidal actions (Wang, 2003; Bureau et al., 2012). Suicide attempts (SA) are self-injurious behaviors with nonfatal consequences; however, they contain a degree of suicidal intention and may result in injury or cause no injury (O’Carroll et al., 1996; Masi et al., 2020). Prior studies suggested that suicidal ideation and suicide attempt are predictive of later suicide death (Hawton and Harriss, 2007; Hawton and O’Connor, 2012; Runeson et al., 2016).

There were many different theories of suicide have been proposed over the past few decades. These theories were based on biological (Oquendo et al., 2014) and sociological approaches (Durkheim, 1897), and psychological approaches. In the psychological approaches that conceptualize suicide as a phenomenon related to the following: psychache (Shneidman, 1993); escape from self-awareness (Baumeister, 1990); hopelessness (Beck et al., 1985); emotion dysregulation (Linehan, 1993); perceived burdensomeness, thwarted belongingness, and capability for suicide (Joiner, 2005; Joiner et al., 2009; Van Orden et al., 2010); “motivational-volitional” model (O’Connor, 2011; O’Connor and Portzky, 2018); and “ideation to action” frameworks (Klonsky and May, 2015; Klonsky et al., 2018) among several others.

The traditional suicide approach treats suicide behavior as a unitary construct, confused the influencing factors of SI and SA, and fails to distinguish the different predictors of SI and SA (Yang et al., 2018). Based on the ideation-to-action framework (Klonsky and May, 2015), the development of suicide ideation and the progression from ideation to attempts as distinct processes with distinct explanations and predictors. Previous studies reported that the combined prevalence of SI among Chinese middle and high school students ranged from 6.76 to 32%, respectively (Chang et al., 2013; Dong et al., 2014; Tan et al., 2016; Li et al., 2019; Xiao et al., 2019; Yang et al., 2019), and the combined prevalence of SA ranged from 1.5 to 9.7%, respectively (Dong et al., 2014; Hu et al., 2015; Liu et al., 2018; Yang et al., 2019; Zhou et al., 2020). A meta-analysis showed that the prevalence of suicide attempts in Chinese mainland middle school students with suicidal ideation was 18% (Shen and Wang, 2020). Given the high prevalence of SI and SA, it is critical to understand the causes of suicide among Chinese adolescents.

As suicide is multifactorial, spanning from psychological, social, cultural, behavioral, and biological factors (Zhang et al., 2010; O’Connor and Nock, 2014). Therefore, the correlational factors of SI and SA should also be multifaceted. To date, many psychological theories of suicide have emerged, and a large number of factors influencing suicidal behavior have been identified. Multiple psychosocial factors are associated with SI and SA among adolescents. The common related factors of SI and SA in adolescents including female gender (Chang et al., 2013; Dong et al., 2014; Liu et al., 2017; Li et al., 2019; Miranda-Mendizabal et al., 2019; Wen et al., 2019; Xiao et al., 2019; Yang et al., 2019; Yuan et al., 2019), socioeconomic condition (Kokkevi et al., 2012; Liu et al., 2017; Begum et al., 2018; Areba et al., 2021), parents educational level (Liu et al., 2018; Wang et al., 2019), depression (Wen et al., 2019; Yang et al., 2019; Yuan et al., 2019; Jung and Cho, 2020; Vargas et al., 2021), hopelessness (May and Klonsky, 2016; Liu et al., 2018; Miranda-Mendizabal et al., 2019), self-esteem (Yang et al., 2013; Hu et al., 2019; Zhu et al., 2019), and impulsive tendency (Tan et al., 2016; Liu et al., 2018; Zhu et al., 2019). The related factors of SI also including bullying (Liu et al., 2013) and life events (Shen et al., 2019). SI was the strongest risk factor for suicide, can increase the likelihood or prevalence of suicide (Liu et al., 2005).

At present, few studies examine SI and SA among adolescents from primary school to high school in China, and these few related studies mainly focus on macro descriptions of SI and SA for this age group. Empirical studies on the influencing factors and mechanisms of SI and SA are scarce. The cumulative contextual risk model suggests that risks do not occur and are not enacted in isolation; they happen cooperatively and relate to one another. Risk factors are co-occurring and interrelated; they affect individuals in a cumulative and superimposed way. The more risk factors an individual experience, the greater the impact they have on the individual. Therefore, it is not the presence or absence of a single risk that affects an individual. The accumulation of risks leads to bad outcomes (Appleyard et al., 2005). Risk factors in families, schools, and social communities pose a serious threat to adolescents’ physical and mental health. Adolescents living in families with inadequate family structure, low socioeconomic status, or a dysfunctional family environment are more likely to have mental health problems (Buehler and Gerard, 2013). Individuals who experience multiple risks simultaneously are more likely to develop psychological disorders than those who experience a single or a small number of risks (Doan et al., 2012). Therefore, some researchers have begun to examine the problem of suicide by referring to the cumulative situational risk model and believe that with the increase in the number of risk factors, the risk of suicide also increases (Roberts et al., 2010). The cumulative ecological risk index was constructed using the generally accepted and widely used modeling methods in the available literature (Buehler and Gerard, 2013; Bao et al., 2014).

Adolescence is a stage of imperfect physical and psychological development, poor emotional processing ability, and susceptibility to various environmental factors, generally leading to higher SI and SA. Due to previous studies’ sample sizes, the findings may not reliably reflect SI and SA among Chinese adolescents. This study’s purpose was as follows: (1) determine the prevalence of suicidality (i.e., SI or SA) among adolescents in a city in Northeast China and (2) identify the correlational factors among adolescents with suicidality. This study was carried out according to the current situation and aims to develop scientific and effective SI and SA protection measures, which may pave the way for follow-up research.

## Materials and Methods

### Participants

This study conducted a cross-sectional online mental health survey in Chinese among 155,242 students from grades 5 to 12 selected from a city in Northeast China.

There were two exclusion criteria: (1) the total score of the Social Desirability Scale (Zhao et al., 2010; Zhao, 2011; Wei et al., 2015) was above 4 (three items, e.g., “I never cry”; “I never swear”; and “I never lie,” ranging from 1 = yes, to 2 = no) and (2) answering time: participants who took fewer than 2 min or more than 60 min were deemed invalid samples. Ultimately, a total of 69,519 valid questionnaires were obtained. Due to the above strict exclusion criteria, this study’s validity rate was 44.8%. Among the participants, there were 34,376 boys and 35,143 girls with no missing data. Table 1 provides the number of students in each grade who participated in the test.

**Table 1:** The number of participants in each grade.

Grade	Total	Boy (*n* = 34,376)	Girl (*n* = 35,143)	Urban (*n* = 36,738)	County (*n* = 10,839)	Rural (*n* = 21,942)
5th	10,287	5,504	4,783	5,267	1713	3,307
6th	8,527	4,401	4,126	3,885	1,541	3,101
7th	9,247	4,838	4,409	4,815	1,371	3,061
8th	9,312	4,536	4,776	5,008	1,306	2,998
9th	10,427	4,963	5,464	5,196	1,420	3,811
10th	7,243	3,416	3,827	4,285	1,067	1891
11th	8,479	3,905	4,574	4,918	1,380	2,181
12th	5,997	2,813	3,184	3,364	1,041	1,592

### Measures

#### Sociodemographic Measures

The sociodemographic measures included age, sex, subjective socioeconomic status (lower, middle, higher), family integrity (nuclear/joint, with joint families being extended and often including multiple generations), family composition (living with two biological parents or any other adults), parents’ education (middle school, high school, college or above), and living area (urban/rural).

#### Suicidal Ideation and Suicide Attempts

Two separate items assessed SI and SA: “During the last year, have you seriously considered attempting suicide?” and “Have you ever actually attempted suicide?” The items were rated as “No” or “Yes.” Affirmative responses for one or both questions above were used to measure SI and SA. Although there were only two items, these are the most widely used measures in suicide research (Herba et al., 2008). The SI and SA measures accorded with Wei and Zhang (2019) study that determined that single-item measures were better if the research aligned with the four following criteria: (1) large-sample survey; (2) single-dimensional construct; (3) specific and clear constructs; and (4) time constraints. To improve the truthfulness of the answers and reduce any discomfort caused by the suicide questionnaire, this study used single-item measures.

#### Self-Esteem

The Rosenberg Self-Esteem Scale (RSES; Rosenberg, 1965) has a total of 10 items and a one-factor structure, ranging from 1 (Strongly disagree) to 4 (Strongly agree), and 4 items (3, 5, 8, 9, 10) had a reversed score; a higher total score indicates a higher level of self-esteem. Four items are used in the present study (e.g., “I feel I have many good qualities”; “I often feel useless”; “I feel that I am a worthy person, at least on the same level as others”; and “In general, I am satisfied with myself”). The present study’s Cronbach’s alpha for this scale was 0.72.

#### Hopelessness

The Beck Hopelessness Scale (BHS; Beck et al., 1974) is a 20-item, self-rated scale consisting of statements that assess thoughts or feelings about the future that the subject rates as true or false (Kong et al., 2007). Eleven items were keyed true, and 9 were false, with a total score of 20 for maximum hopelessness. Two items were selected from the Beck Hopelessness Scale (“My future seems hopeless” and “I feel useless”), each of which was rated on a 5-point Likert scale (1 = Strongly Disagree, 2 = Disagree, 3 = Uncertain, 4 = Agree, and 5 = Totally Agree) to indicate intensity. The present study’s Cronbach’s alpha for this scale was 0.84.

#### The Adolescent Self-Rating Life Events Checklist

The Adolescent Self-Rating Life Events Checklist (ASLEC; Xin and Yao, 2015) was used to assess whether adolescent life events occur and the degree of their impact; it includes 27 items. The subjects were asked to answer whether the events presented in the text have occurred either to themselves or their families in the past 12 months. If an event did occur, it was selected according to its degree of impact, which is classified into five levels: 1 = no impact, 2 = mild, 3 = moderate, 4 = severe, and 5 = very severe. In this study, there were 6 options, since 0 = no occurrence was also a choice; the total score reflected negative life events’ degree of impact on participants. Five items were selected in this study. The Cronbach’s alpha for these five items in the present study was 0.74.

#### The Barratt Impulsiveness Scale

The Barratt Impulsivity Scale (BIS-11) is a commonly used impulsivity assessment tool in psychiatric and psychological research (Yang et al., 2007). Three items were selected from the Barratt Impulsiveness Scale (“I often do things without thinking”; “I often do what occurs to me without considering the consequences”; “I’m always excited about new ideas, but I rarely think about possible difficulties”), each of which was rated on a 5-point Likert scale (1 = Strongly Disagree, 2 = Disagree, 3 = Uncertain, 4 = Agree, and 5 = Totally Agree) to indicate intensity. The Cronbach’s alpha for these 3 items in the present study was 0.76.

#### The Cumulative Risk Index

The cumulative risk index was constructed using the generally accepted and widely used modeling methods in the available literature (Buehler and Gerard, 2013; Bao et al., 2014). According to Gerard and Buehler’s (2004) previous cumulative risk study, the risk factors should be record into dichotomous variables. The dichotomous variables value is assigned to 1(having risks), and the remaining values are assigned to 0(no risk).

#### Survey Procedure

Data were collected through administering the questionnaires online in September of 2020. After depositing all questions in Wenjuanxing platform, a shareable link was generated. Informed consent was obtained from school administrators, teachers, students, and parents before data collection by Educational administration. Educational administration staffs verbally informed respondents about the study and shared that participation was optional. Students were informed that they could voluntarily participate the survey and withdraw at any time. All personal information collected during the survey would be kept anonymous. The survey used a self-reported questionnaire that included two sections: sociodemographic characteristics and mental health self-rating scales. All instruments were administered in Chinese. The School of Psychology of Northeast Normal University conducted this survey and obtained permission to survey all included schools from their administrators. The study was approved by the Research Ethics Committee of Northeast Normal University School of Psychology.

### Statistical Analysis

Data were analyzed using Microsoft Excel 2010 and SPSS software version 22.0. Microsoft Excel was used to edit, sort, and code data; then, the Excel file was imported into SPSS software. Descriptive statistics (frequencies, percentages, means, and standard deviation) and some analyses (i.e., chi-square tests, univariable logistic regression, and multivariable logistic regression) were performed using SPSS software. Logistic regression was performed with a 95% confidence interval to determine significant associations between the categorical dependent and independent variables. Analyses were univariate, yielding crude odds ratios (CORs), followed by multivariable analyses with predictors (only significant variables from unadjusted models) combined in the models and yielding adjusted odds ratios (AORs). The association of variables was considered statistically significant if the two-sided *p* < 0.05.

## Results

### Common Method Bias Test

The data were collected using the self-report method, which will result in artificial covariation between the dependent and independent variables. Therefore, a test was required for common method bias. In this study, the Harman single-factor test was used for the common method bias test (Zhou and Long, 2004). The results showed that the variance explained by the first principal factor was 19%, which is less than 40%, indicating that common method bias was not significant.

### Sample Characteristics and Prevalence of SI and SA

The prevalence of SI and SA among Chinese adolescents was 13 and 4.8%, respectively. A total of 69,519 respondents were recruited into the final analyses; 49.4% were male and ranged from grades 5 to 12, 52.8% lived in cities, and 89.3% lived with parents. Most were from nuclear families (84%), and the majority were from the middle SES group (73.1%). Other demographic characteristics are reported in Table 2.

**Table 2:** Demographic characteristics of participants and regression analysis (unadjusted estimates) by SI and SA of the participants.

Characteristics	Total *N* (%)	SI				SA			
		*n* (%) or M ± SD	*χ*^2^/*t*-value	*p*-value	Unadjusted OR (95% CI)	*n* (%)or M ± SD	*χ*^2^/*t*-value	*p*-value	Unadjusted OR (95% CI)
**Sex**			683.12	<0.001			442.65	<0.001	
Male	34,376 (49.4)	3,306 (9.6)			1.00 [Reference]	1,065 (3.1)			1.00 [Reference]
Female	35,143 (50.6)	5,722 (16.3)			1.83 (1.75,1.91)	2,291 (6.5)			2.18 (2.02,2.35)
**Grade**			1639.96	<0.001			580.04	<0.001	
5th	10,287 (14.8)	417 (4.1)			1.00 [Reference]	151 (1.5)			1.00 [Reference]
6th	8,527 (12.3)	646 (7.6)			1.94 (1.71,2.20)	247 (2.9)			2.00 (1.63,2.46)
7th	9,247 (13.3)	930 (10.1)			2.65 (2.35,2.98)	375 (4.1)			2.84 (2.34,3.44)
8th	9,312 (13.4)	1,503 (16.1)			4.56 (4.07,5.09)	618 (6.6)			4.77 (3.99,5.71)
9th	10,427 (15.0)	1,862 (17.9)			5.15 (4.61,5.743)	778 (7.5)			5.41 (4.54,6.46)
10th	7,243 (10.4)	1,113 (15.4)			4.29 (3.82,4.83)	386 (5.3)			3.78 (3.12,4.57)
11th	8,479 (12.2)	1,542 (18.2)			5.26 (4.70,5.89)	490 (5.8)			4.12 (3.42,4.57)
12th	5,997 (8.6)	1,015 (16.9)			4.82 (4.28,5.43)	311 (5.2)			3.67 (3.02,4.47)
**Residence**			191.27	<0.001			28.34	<0.001	
City	36,738 (52.8)	5,342 (14.5)			1.00 [Reference]	1918 (5.2)			1.00 [Reference]
Town	10,839 (15.6)	1,361 (12.6)			0.84 (0.79,0.90)	502 (4.6)			0.88 (0.79,0.98)
Rural	21,942 (31.6)	2,325 (10.6)			0.69 (0.66,0.73)	936 (4.3)			0.81 (0.75,0.88)
**Family composition**			111.88	<0.001			81.57	<0.001	
Live with two biological parents	62,113 (89.3)	7,777 (12.5)			1.00[Reference]	2,841 (4.6)			1.00 [Reference]
Other (Not live with two biological parents)	7,406 (10.7)	1,251 (16.9)			1.42 (1.33,1.51)	515 (7.0)			1.56 (1.42,1.72)
**Family integrity**									
Nuclear	58,379 (84)	6,849 (11.7%)	507.32	<0.001	1.00 [Reference]	2,454 (4.2%)	308.65	<0.001	1.00 [Reference]
Other	11,140 (16)	2,179 (19.6%)			1.83 (1.74,1.93)	902 (8.1%)			2.01 (1.86,2.17)
**Father’ s education level**									
≤ High school degree	58,000 (83.4)	7,624 (13.1)	7.78	<0.01	1.09 (1.03,1.16)	2,864 (4.9)	9.29	<0.01	1.16 (1.06,1.28)
≥ College degree	11,519 (16.6)	1,404 (12.2)			1.00 [Reference]	492 (4.3)			1.00 [Reference]
**Mother’ s education level**									
≤High school degree	57,720 (83)	7,598 (13.2%)	9.45	<0.01	1.10 (1.04,1.17)	2,859 (5.0%)	11.71	<0.01	1.19 (1.08,1.31)
≥College degree	11,799 (17)	565 (12.1%)			1.00 [Reference]	173 (4.2%)			1.00 [Reference]
**Subjective SES**									
Lower	8,364 (12)	469 (19.5%)	376.43	<0.001	1.81 (1.70,1.92)	635 (7.6%)	201.94	<0.001	1.88 (1.71,2.06)
Higher	10,350 (14.9)	1,129 (13.3%)			1.14 (1.07,1.21)	592 (5.7%)			1.39 (1.26,1.52)
Middle	50,805 (73.1)	6,020 (11.8%)			1.00 [Reference]	2,129 (4.2%)			1.00 [Reference]
**Feeling Meaningless in Life**									
Yes	14,489 (20.8)	7,358 (50.8%)	23140.95	<0.001	32.97 (31.09,34.96)	2,999 (20.7%)	10035.09	<0.001	39.75 (35.75,44.69)
No	55,030 (79.2)	1,670 (3.0%)			1.00 [Reference]	357 (0.6%)			1.00 [Reference]
**Depression**									
Yes	17,512 (25.2)	7,101 (40.5%)	15738.16	<0.001	17.73 (16.78,18.72)	2,915 (16.6%)	7116.32	<0.001	23.35 (21.09,25.85)
No	52,007 (74.8)	1,927 (3.7%)			1.00 [Reference]	441 (0.8%)			1.00 [Reference]
**Bullying Perpetrator**									
Yes	1871 (2.7)	641 (34.3%)	770.05	<0.001	3.68 (3.34,4.06)	394 (21.1%)	1102.48	<0.001	5.83 (5.18,6.55)
No	67,648 (97.3)	8,387 (12.4%)			1.00 [Reference]	2,962 (4.4%)			1.00 [Reference]
**Autonomy Parenting**									
Yes	29,469 (42.4)	2,321 (7.9%)	1182.18	<0.001	1.00 [Reference]	727 (2.5%)	620.34	<0.001	1.00 [Reference]
No	40,050 (57.6)	6,707 (16.7%)			2.35 (2.24,2.47)	2,629 (6.6%)			2.78 (2.56,3.02)
**Self-esteem (M ± SD)**	69,519	2.53 ± 0.61	−81.59	<0.001	0.63 (0.62,0.63)	2.19 ± 0.63	−59.76	<0.001	0.61 (0.59,0.62)
**Hopelessness (M ± SD)**	69,519	6.35 ± 2.16	87.59	<0.001	1.73 (1.71,1.75)	6.67 ± 2.24	62.40	<0.001	1.78 (1.75,1.82)
**Stressful Life Events (M ± SD)**	69,519	11.52 ± 4.64	83.81	<0.001	1.24 (1.23,1.24)	12.44 ± 5.09	55.06	<0.001	1.24 (1.23,1.25)
**Impulsivity (M ± SD)**	69,519	8.67 ± 2.71	25.31	<0.001	1.12 (1.11,1.13)	8.91 ± 2.87	17.93	<0.001	1.15 (1.14,1.17)

SI, Suicide ideation; SA, Suicide attempts. ^*^p < 0.05; ^**^p < 0.01; ^***^p < 0.001.

**Figure 1:**
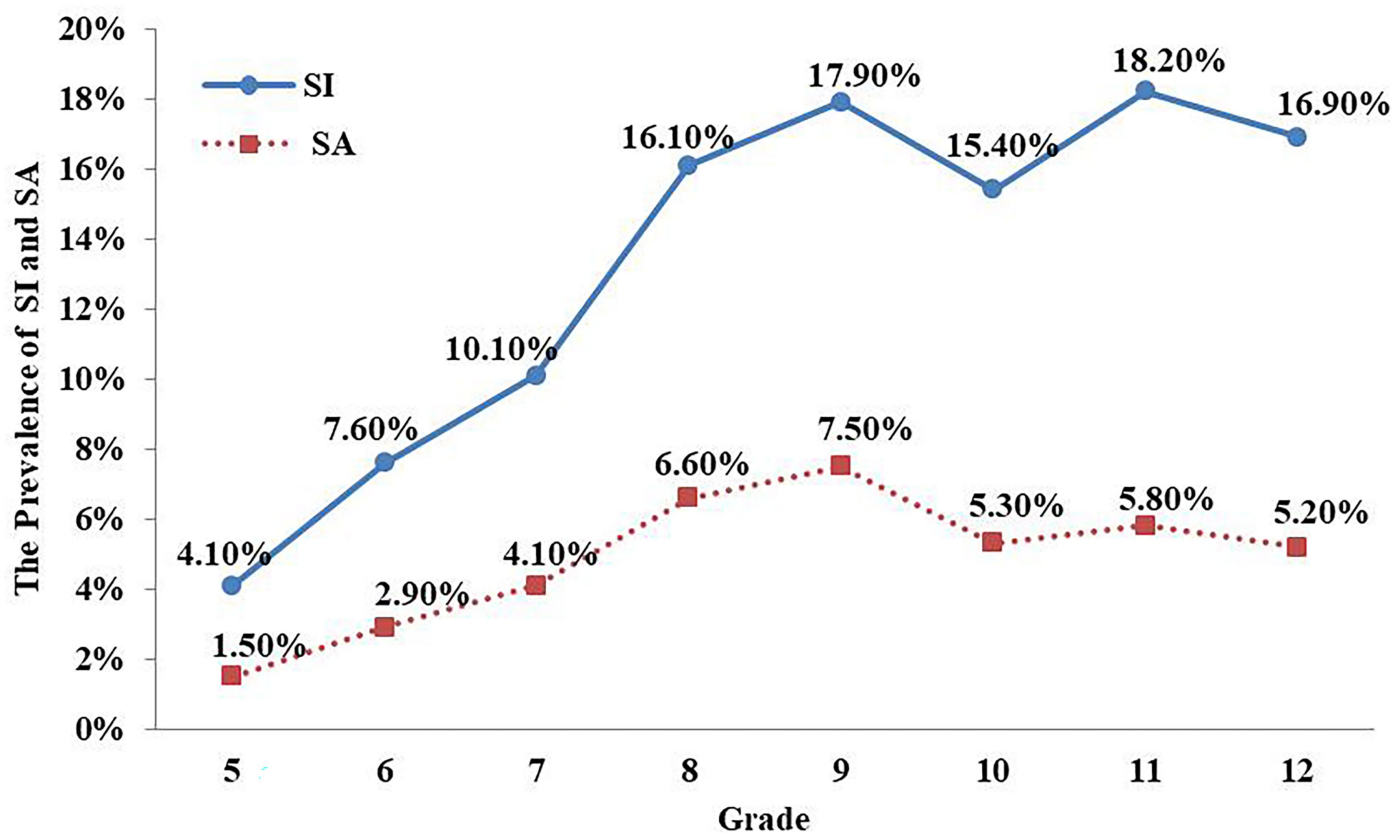
The prevalence of SI and SA between different grades.

As Table 2 shows, being female, of advanced age, having a low level of father and mother’s education, being from a non-nuclear family, having a lower subjective SES, feeling meaningless in life, experiencing depression, being bullied by a perpetrator, being subjected to parents’ poor rearing pattern, having low esteem, feeling hopeless, experiencing negative life events, and being characterized by impulsivity were all significantly associated with certain types of SI and SA (all *p* < 0.05). As Figure 1 shows, the prevalence of SI and SA that students reported increased as their grade-level increased.

Univariable regression analysis was performed to assess potential risk and protective factors for SI and SA (Table 2). Females were 1.83 and 2.18 times more likely to have SI and SA than males. Additional risk factors for SI and SA were as follows: individuals with lower and higher SES vs. middle SES; individuals with vs. without a nuclear family; individuals with vs. without parents’ higher educational level; individuals with vs. without feeling meaninglessness in life; individuals with vs. without depression; individuals with vs. without a bullying perpetrator; individuals with vs. without parents’ autonomous parenting styles; individuals with vs. without higher hopelessness scores and impulsivity scores; and individuals with vs. without experiencing a serious impact from life events.

Respondents with lower self-esteem scores were 0.63 and 0.61 times more likely to have SI and SA, respectively, than those with higher self-esteem scores. Additional protective factors mitigating SI and SA were reflected in individuals living in rural vs. urban areas.

### Multivariable Logistic Regression Analysis

Multivariable analyses with predictors (only significant variables from the unadjusted model) entered jointly in the model revealed that most factors remained significant (see Table 3). Table 3 shows the multivariable logistic regression analysis results.

**Table 3:** Multivariable logistic regression analysis of risk factors for SI and SA.

Characteristics	SI Adjusted model		SA Adjusted model	
	*AOR* (95%CI)	*p*-value	*AOR* (95%CI)	*p*-value
**Sex**				
Male	Reference		Reference	
Female	1.58 (1.49–1.68)	<0.001	1.74 (1.60–1.89)	<0.001
**Grade**				
5th	Reference		Reference	
6th	1.22 (1.05–1.43)	<0.05	1.10 (0.87–1.39)	0.41
7th	1.33 (1.15–1.54)	<0.001	1.26 (1.01–1.57)	<0.05
8th	1.54 (1.34–1.77)	<0.001	1.30 (1.50–1.61)	<0.05
9th	1.58 (1.38–1.82)	<0.001	1.35 (1.10–1.66)	<0.01
10th	1.54 (1.33–1.78)	<0.001	1.19 (0.96–1.49)	0.12
11th	1.52 (1.32–1.75)	<0.001	1.00 (0.81–1.24)	0.98
12th	1.29 (1.12–1.51)	<0.01	0.88 (0.70–1.10)	2.63
**Residence**				
City	Reference		Reference	
Town	0.87 (0.79–0.94)	<0.01	0.95 (0.84–1.07)	0.37
Rural	0.68 (0.63–0.73)	<0.001	0.90 (0.81–0.99)	0.03
**Family composition**				
Live with two biological parents	Reference		Reference	
Other (Not live with two biological parents)	1.01 (0.92–1.12)	0.79	0.97 (0.85–1.09)	0.59
**Family integrity**				
Nuclear	Reference		Reference	
Other	1.22 (1.13–1.33)	<0.001	1.26 (1.13–1.40)	<0.001
**Father’s education level**				
≤ High school degree	0.99 (0.89–1.11)	0.95	1.03 (0.89–1.20)	0.68
≥ College degree	Reference		Reference	
**Mother’ s education level**				
≤ High school degree	1.03 (0.92–1.15)	0.59	1.04 (0.89–1.20)	0.65
≥ College degree	Reference		Reference	
**Subjective SES**				
Lower	1.05 (0.97–1.14)	0.22	1.00 (0.89–1.12)	0.94
Higher	1.26 (1.16–1.37)	<0.001	1.61 (1.44–1.81)	<0.001
Middle	Reference		Reference	
**Feeling Meaningless in Life**				
Yes	9.24 (8.62–9.90)	<0.001	8.66 (7.62–9.85)	<0.001
No	Reference		Reference	
**Depression**				
Yes	4.09 (3.83–4.37)	<0.001	4.24 (3.78–4.76)	<0.001
No	Reference		Reference	
**Bullying Perpetrator**				
Yes	1.67 (1.46–1.91)	<0.001	2.73 (2.35–3.17)	<0.001
No	Reference		Reference	
**Autonomy Parenting**				
Yes	Reference		Reference	
No	1.31 (1.23–1.40)	<0.001	1.29 (1.17–1.42)	<0.001
**Self-esteem**	0.91 (0.89–0.93)	<0.001	0.88 (0.86–0.90)	<0.001
**Hopelessness**	1.09 (1.07–1.11)	<0.001	1.10 (1.07–1.13)	<0.001
**Stressful Life Events**	1.07 (1.06–1.07)	<0.001	1.06 (1.05–1.07)	<0.001
**Impulsivity**	0.99 (0.98–1.00)	0.12	1.01 (0.99–1.03)	0.12

CI: Confidence interval. AOR: Adjusted Odds Ratio. ^*^p < 0.05; ^**^p < 0.01; ^***^p < 0.001.

Multivariable analyses with predictors (only significant variables from the unadjusted model) entered jointly in the model revealed that most factors remained significant, with residence, family composition, parents’ education level, lower SES, and impulsivity losing statistical significance (see Table 3). For SI, the model was statistically significant, *χ*^2^ = 23325.19, *df* = 24, *p* < 0.001 (Cox and Snell^2^ = 0.28; Nagelkerke *R*^2^ = 0.53). For SA, the model was statistically significant, *χ*^2^ = 10406.79, *df* = 24, *p* < 0.001 (Cox and Snell^2^ = 0.14; Nagelkerke *R*^2^ = 0.43). Results show that, compared with the reference group, female gender is a significant predictor of the SI and SA group. Living in city areas increased participants’ odds of belonging in the SI groups. The co-occurring SI/SA group is more likely to be affected by family integrity, subjective SES, feeling meaningless in life, depression, bullying, autonomy parenting, self-esteem, hopelessness, and stressful life events. Grade is a significant predictor of SI and SA.

### The Relationship Between Cumulative Risk Factors and SI and SA

As Table 3 shows, after controlling for other variables for SI and SA—female, non-nuclear family, higher subjective socioeconomic status, meaningless in life, depression, bullying perpetrator, negative parental rearing styles, higher self-esteem scores, hopelessness, and stressful life events—the 10 risk factors are still significant. And for SI, the residence of adolescents is significant too. Therefore, according to the cumulative risk model, the 11 and 10 projects are, respectively, synthesized into a psychological accumulation of risk factors to examine whether the prevalence of SI and SA among adolescents would continue growing as psychological cumulative risk scores increase. Since the measurement methods for the 11 risk factors are inconsistent, according to Gerard and Buehler’s (2004) previous cumulative risk study, the 11 risk factors should be record into dichotomous variables. The dichotomous variables value is assigned to 1(having risks), and the remaining values are assigned to 0(no risk). Due to the prevalence of SI and SA among Grade 8–12 students was relatively stable. The relationship between the cumulative risk factors and suicidality (SI and SA) was analyzed by the students from Grade 8–12.

**Figure 2:**
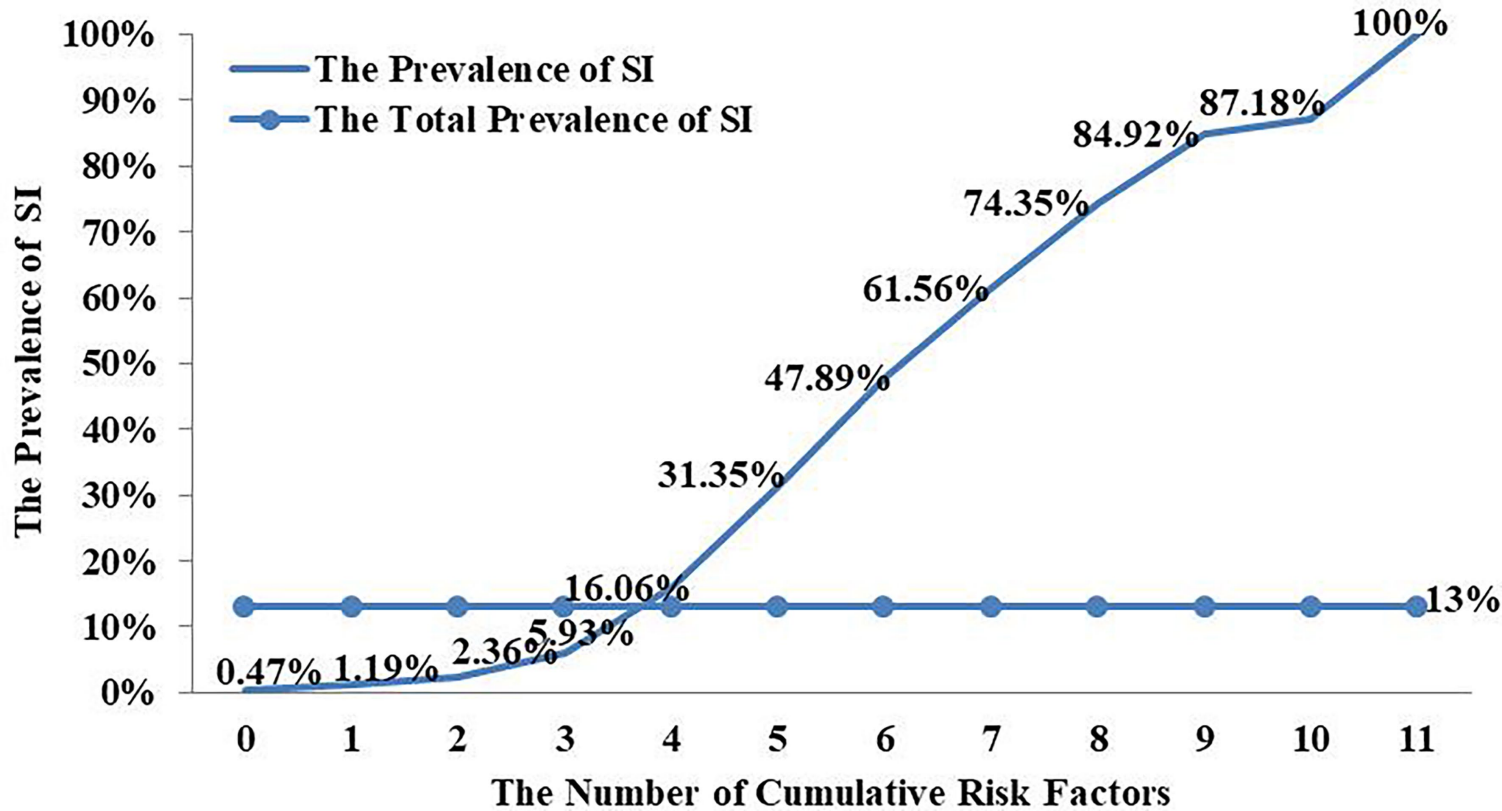
SI risk among Chinese adolescents from Grade 8 to 12 under different Cumulative risk factors.

**Figure 3:**
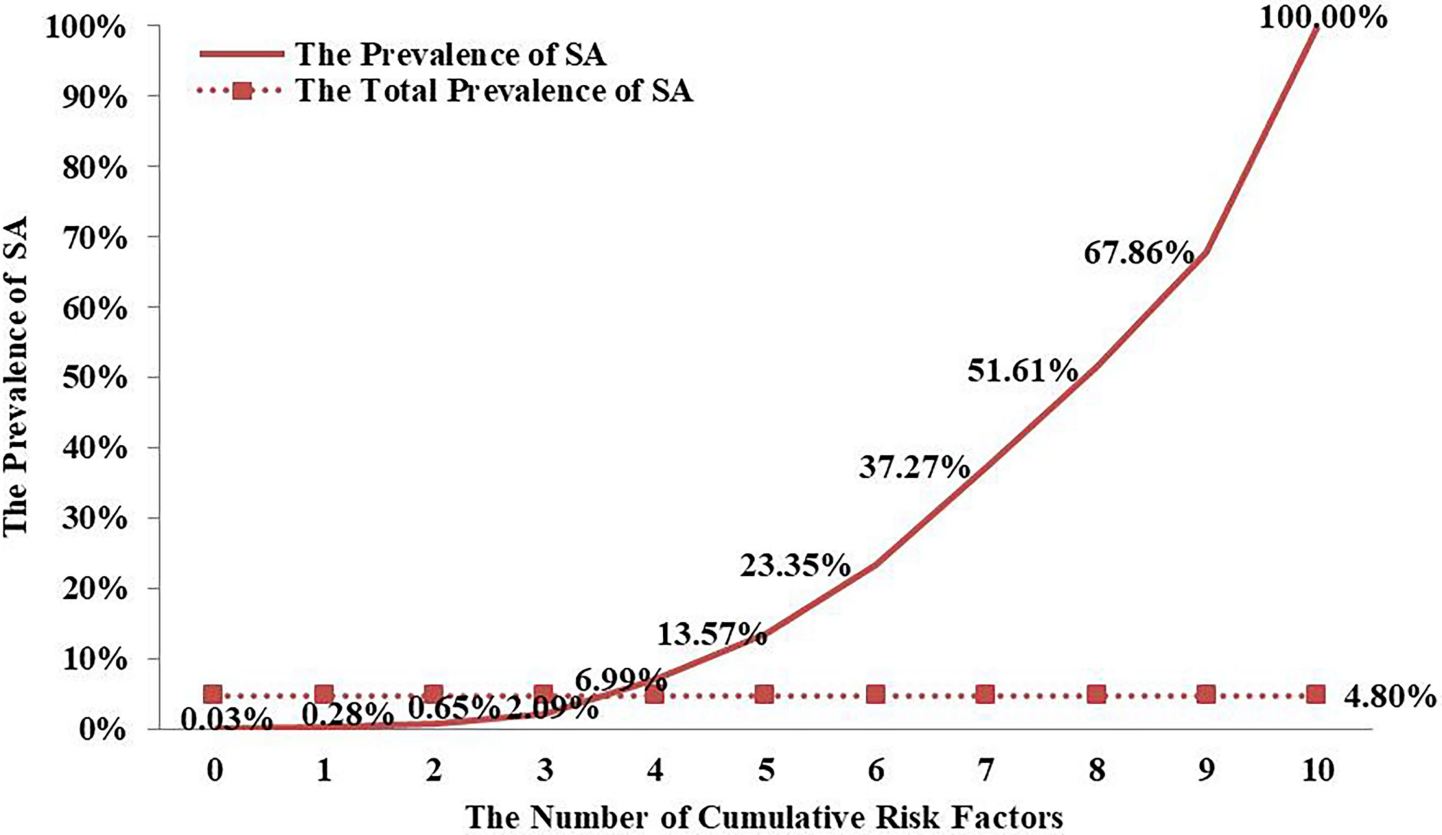
SA risk among Chinese adolescents from Grade 8 to 12 under different Cumulative risk factors.

As Figures 2, 3 show, as cumulative risk factors increase, the prevalence of SI and SA among Chinese adolescents shows an increasing trend.

## Discussion

The major findings are (1) SI and SA were prevalent and increased before Grade12 in Chinese adolescents; (2) SI and SA were more prevalent in girls than in boys; and (3) SI and SA shared similar multiple risk factors.

In this study, a total of 150,000 primary and middle school students from grades 5 to grade 12 in a city from Northeast China were surveyed. According to the two exclusion criteria, more than 69,000 effective responses were obtained, which can comprehensively reflect the current situation of SI and SA among primary and middle school students in a city in Northeast China. In the sample of Chinese adolescents, 13% had ever thought about it in the last year, and 4.8% had ever attempted suicide in the last year. The rates of SI and SA are comparable to those reported in previous studies of Chinese and Western adolescents (Dong et al., 2014; Hu et al., 2015; Liu et al., 2017; Li et al., 2019; Lim et al., 2019; Yang et al., 2019; Forster et al., 2020). For example, in a meta-analysis of suicide-related behaviors in Chinese adolescents, the authors found that the prevalence rates of SI and SA were 17.7 and 2.7% (Dong et al., 2014). And a meta-analysis conducted in 2018 showed that the prevalence rates in the past year globally were 14.2 and 4.5%, respectively (Lim et al., 2019). These studies consistently discovered that teenagers have high suicidal intentions, confirming that adolescent suicide problems require researchers’ attention.

Sex was significantly associated with SI in SA. Similar to findings from Chinese adolescents (Chang et al., 2013; Dong et al., 2014; Liu et al., 2017; Li et al., 2019; Wen et al., 2019; Xiao et al., 2019; Yang et al., 2019; Yuan et al., 2019), girls were found an elevated level of SI and SA. For example, in a meta-analysis of gender differences in suicidal behavior in adolescents and young adults, the authors found that the prevalence rates in females are almost double those in males for SA (Miranda-Mendizabal et al., 2019). The reasons for this result maybe as following: the significant developmental lag between hormonal changes and emotional status leaves adolescents prone to increased affective disorders (Stoep et al., 2009); female had high score of passive coping (Yao et al., 2014).

This study found a significant relationship between suicidality (SI and SA) and socioeconomic status: students from higher SES families had more SI and SA than those from lower SES families, similar to prior findings among adolescents (Begum et al., 2018; Irish and Murshid, 2020).

Consistent with previous studies, the logistic regression results show that students with high SI and SA have experienced more negative life events (Shen et al., 2019), greater feelings of depression (Thompson et al., 2005; Liu et al., 2013; Park, 2017; Jung and Cho, 2020), and more hopelessness (Thompson et al., 2005; Tan et al., 2016); they have also engaged in bullying behavior (Liu et al., 2013). Therefore, it is necessary to provide comprehensive and timely intervention measures after detecting SI and SA among adolescents. These students should be taught how to appropriately handle stress, difficulties, and setbacks. In addition, more corresponding lectures and group counseling activities should be carried out during their study careers. In previous studies, a decrease in self-esteem led to SI (Ozdemiroglu et al., 2017; Park, 2017). High self-esteem can help individuals better adjust their behavior and mood, thus reducing the occurrence of SI and SA. Low self-esteem can affect individuals’ ability to adapt to the environment, which results in greater SI and SA (Yang et al., 2013; Ozdemiroglu et al., 2017; Park, 2017; Hu et al., 2019). Depression is one of the most important vulnerability factors in individuals, and it impacts SI and SA through the negative cognition process of despair (Jung and Cho, 2020).

Compared with the single risk, the cumulative risk is more in line with the reality of human life and will cause more serious harm to individual development (Appleyard et al., 2005; Evans et al., 2013). This study found that female, non-nuclear family, higher subjective socioeconomic status, meaningless in life, depression, bullying perpetrator, negative parental rearing styles, higher self-esteem scores, hopelessness, and stressful life events were all high-risk predictors of SI and SA in adolescents. As the results show as cumulative risk factors increase, the prevalence of SI and SA among Chinese adolescents shows an increasing trend. Studies have shown that individual exposed the more risk factors, the higher the risk of suicidality (Roberts et al., 2010; Ge and Liu, 2018). The Cumulative risk index was very necessary. As in the context of Chinese culture, measure students’ SI and SA use the Structured and Standardized questionnaires would let their parents feel that the researchers may induce students’ suicidal intentions. It was a relative worthwhile method to use the Cumulative risk index to investigate students’ the risky of SI and SA. Clearly, that proved the validity of the cumulative risk index. If these high-risk factors can be identified sooner, corresponding measures can be taken early to intervene and avoid unfortunate events.

## Implications

This study has significant implications for practice and research. The practical application value of this study is manifested in the following two aspects: first, it can reduce the burden of participants to answer; second, the results based on the large sample can make up for the instability of the survey results. For researchers and educators, as suicide is a global public health event and social problem, it is not sufficient to identifying risk factors for suicide. Because the interaction of multiple factors often increases the risk of suicide, and some combinations may be particularly dangerous. Therefore, it is essential to investigate the interactions between these different contributors to understand how they interact to confer risk. In order to improve the accuracy of suicide risk identification, we made a cumulative risk index, because an increase in the index increases the risk of suicide. With the increase in the number of cumulative risks, the risk of SI and SA also increased. So the cumulative risk index was very valuable. For adolescents, the total prevalence of SI and SA among primary and middle school students was high. Preventive measures could be implemented according to the risk factors (such as feeling meaningless in life, depression, hopelessness, stressful life events, and so on). The adolescents who with higher cumulative risk index scores should be noted.

## Limitations

First, SI and SA measurements in this study were obtained by simple “yes” or “no” answers, which have certain limitations and cannot sufficiently show the severity or developmental stage of SI and SA. Therefore, further studies should investigate the developmental stages of SI and SA in detail and develop interventions adapted to each according to the different developmental stages.

Second, some of the factors influencing SI and SA were self-reported. This study does not provide an in-depth combination of corresponding qualitative research and in-depth interviews with adolescents who have experienced SI and attempted suicide, nor does it give an accurate and in-depth assessment of the actual situation or detailed psychological changes in SI and SA.

Third, most of the previous studies were cross-sectional studies. Only longitudinal studies can better reflect the role of cumulative risk factors in predicting SI and SA. Therefore, further studies should investigate SI and SA by longitudinal studies.

## Data Availability Statement

The original contributions presented in the study are included in the article/supplementary material, further inquiries can be directed to the corresponding author.

## Ethics Statement

The studies involving human participants were reviewed and approved by the Research Ethics Committee of Northeast Normal University School of Psychology. Written informed consent to participate in this study was provided by the participants’ legal guardian/next of kin.

## Author Contributions

YY contributed to the research design, performed the analysis, and drafted the manuscript. XG provided useful suggestions in the research design stage and contributed to organizing the database. All authors contributed to the article and approved the submitted version.

## Funding

This work was supported by the Research Program Funds of the Collaborative Innovation Center of Assessment toward Basic Education Quality at Beijing Normal University (2021-03-002-BZPK01).

## Conflict of Interest

The authors declare that the research was conducted in the absence of any commercial or financial relationships that could be construed as a potential conflict of interest.

## Publisher’s Note

All claims expressed in this article are solely those of the authors and do not necessarily represent those of their affiliated organizations, or those of the publisher, the editors and the reviewers. Any product that may be evaluated in this article, or claim that may be made by its manufacturer, is not guaranteed or endorsed by the publisher.
